# 3D Printing as a Promising Tool in Personalized Medicine

**DOI:** 10.1208/s12249-020-01905-8

**Published:** 2021-01-17

**Authors:** Vanessa Marcia Vaz, Lalit Kumar

**Affiliations:** Department of Pharmaceutics, Manipal College of Pharmaceutical Sciences, Manipal Academy of Higher Education, Manipal, Udupi, Karnataka 576 104 India

**Keywords:** 3D printing, personalized medicine, dosage forms, polypill, clinical practice

## Abstract

Personalized medicine has the potential to revolutionize the healthcare sector, its goal being to tailor medication to a particular individual by taking into consideration the physiology, drug response, and genetic profile of that individual. There are many technologies emerging to cause this paradigm shift from the conventional “one size fits all” to personalized medicine, the major one being three-dimensional (3D) printing. 3D printing involves the establishment of a three-dimensional object, in a layer upon layer manner using various computer software. 3D printing can be used to construct a wide variety of pharmaceutical dosage forms varying in shape, release profile, and drug combination. The major technological platforms of 3D printing researched on in the pharmaceutical sector include inkjet printing, binder jetting, fused filament fabrication, selective laser sintering, stereolithography, and pressure-assisted microsyringe. A possible future application of this technology could be in a clinical setting, where prescriptions could be dispensed based on individual needs. This manuscript points out the various 3D printing technologies and their applications in research for fabricating pharmaceutical products, along with their pros and cons. It also presents its potential in personalized medicine by individualizing the dose, release profiles, and incorporating multiple drugs in a polypill. An insight on how it tends to various populations is also provided. An approach of how it can be used in a clinical setting is also highlighted. Also, various challenges faced are pointed out, which must be overcome for the success of this technology in personalized medicine.

## INTRODUCTION

The current scenario of medical treatment is centered on the paradigm “one size fits all” where most patients receive same drugs at the same doses and frequencies as others (1). It came to light that this theory of “one size fits all” does not hold up in all treatments. Administration of the same active ingredient at the same dose to different individuals has shown varied responses. The response might be exaggerated and linked with adverse drug reactions (ADRs) or too weak, with insufficient or no pharmacological effects. Both these situations can be succeeded by added patient complications (2). This leads to the initiation of personalized medicines where medications are tailored to patients or designed more particularly for them as part of a group of genetically, physiologically or pathologically similar patients (3). With a mantra of “one size does not fit all”, its goal is to dispense the best drug at the best dose, for the precise indication of the patient, at the correct time (1). Personalized medicine promises are more precise medications. These are more safe and efficacious, improve patient compliance, and are cost-effective (4).

Three-dimensional printing, also called 3D printing or additive manufacturing, involves the deposition of material in a layer upon layer manner to gradually construct a solid model. It uses a computer-aided design (CAD) software which transfers the necessary signals to a 3D printer, which then converts the computerized digital model into two-dimensional (2D) sections, which generates solid layers to build up the required objects (5). It has been widely used in various industries, from automobile and aerospace to biomedical and pharmaceutical industry. It is also being used in the construction of buildings, entertainment, fashion industry, art, and jewelry. In the pharmaceutical industry, it has been employed for the fabrication of various pharmaceutical products like controlled release tablets, polypills, oro-dispersible films, gastrofloating tablets, self-emulsifying drug delivery systems, microneedles, and transdermal patches (6). The various printing technologies include inkjet printing method, binder jet printing method, fused deposition method, selective laser sintering, stereolithography, and pressure-assisted microsyringe (7).

With 3D printing, pharmaceuticals have the potential to bring in a major change in the design, use, and manufacture of different pharmaceutical products. Conventional manufacturing processes, although cost-effective, might call for rigorous labor and can be time-consuming when it comes to large-scale production. In conventional manufacturing processes, also, the doses cannot be manipulated easily according to the patient needs. 3D printing can transform healthcare through personalized medicine, thus improving patient compliance by tailoring the medication to the patient. This can be achieved through on-demand manufacturing in clinical settings to offer the best medical care (8).

There is a considerable amount of literature review on 3D printing and its application in drug delivery. However, there are only limited number of articles which explain the various technologies involved in 3D printing and their applications in pharmaceuticals, along with their use in personalized medicine and their ability to tend to various populations.

## HISTORY OF 3D PRINTING

The earliest research in 3D printing dates back to the late 1970s, which saw various patents regarding the techniques of computer-aided additive manufacture, employing different platforms (9). In the mid-1980s, Charles (Chuck) Hull, also regarded as the pioneer of this technology, invented and patented stereolithography (SLA), which is one of the major technologies in 3D printing. This process involved resins which were polymerized using UV light to obtain the desired object. These SLA printers were then commercialized by 3D Systems, which was founded by Hull (10,11).

In 1986, Carl Deckard, a university student of Texas, developed another technology known as Selective laser sintering which employed laser to fuse powder together. This was followed by another patent for fused deposition modeling in 1989 by Scott and Lisa Crump at the company Stratasys. This process involved heating and extruding plastic or metal using nozzle. Later in 1989, Emanuel Sachs and his associates at MIT developed “three-dimensional printing techniques” which employed binding solution extruded on a powder bed by modifying the inkjet printer. This later came to be known as the “binder jetting” method. Hans Langer in 1989 focused on direct metal laser sintering which utilized laser, to produce 3D objects using computer models (10,12).

Various initiatives were carried out so that people could obtain the low cost and non-proprietary printers. Replicating rapid prototyping (Rep Rap) project was founded and carried out by Andrew Bowyer of the University of Bath which involved developing 3D printers that produce most of its own components, which then grew widely with several collaborations (9).

Since its invention and the use of 3D printing technology has expanded to various fields. Initially, clinical applications in healthcare were for surgical planning and guidance and to produce implants. Implants loaded with active pharmaceutical ingredients were also developed with a good potential for personalization (13). 3D printing was also used for clinical educational purposes (14). 3D printing has now entered the pharmaceutical sector, where it has been used to develop various dosage forms. In 2015, the very first 3D printed drug, Spritam (Levetiracetam), a prescription drug for epilepsy developed by Aprecia Pharmaceuticals was approved by the FDA. This was manufactured using the binder jet printing method and is capable of rapid oral dissolution because of its highly porous structure (15).

## 3D PRINTING TECHNOLOGIES USED IN PHARMACEUTICAL DEVELOPMENT

### Inkjet Printing Method

In general, inkjet printing describes systems which use pattern generating devices to digitally control and place small liquid drops on a substrate. In pharmaceuticals, appropriate mixtures of drug, along with suitable excipients (known as ink) are deposited as small drops in a layer wise fashion on a suitable substrate. Continuous inkjet printing (CIJ) and drop on demand (DoD) are the two main inkjet printing platforms (16,17).

#### Continuous Inkjet Printer

As the name suggests, continuous inkjet printers eject stream of liquid droplets on a substrate continuously, even when the droplets are not necessary. Here, a pressure wave is generated into the ink stream, which breaks up the ink into uniform sized droplets by means of vibration of the nozzle and then ejects the droplets out of the nozzle. Since it ejects droplets continuously, this technology leads to the wastage of ink. The advantages of this printing technology include high-speed continuous droplet generation, due to which the nozzle does not get clogged easily. Disadvantages include low-resolution and expensive maintenance (18).

#### Drop-on-Demand Inkjet Printer

In these printers, drops of liquid are ejected from the printhead due to a trigger signal, only when it is necessary and the drops are deposited onto a substrate. This type of printer typically contains many nozzles (100–1000, but specialized printheads compose of only one). Contrasting the continuous inkjet printer where ejection of droplets is due to the external pressure, drop-on-demand inkjet printers have the drops kinetic energy derived from sources close to each nozzle and located within the printhead (19). This technology is relatively simple, offers high precision and is low cost. It has the potential to deposit small drops of controllable sizes and also produces them with good placement accuracy. It also minimizes the wastage of drugs. Hence, it is favored over continuous inkjet printing for printing applications (20,21).

Drop-on-demand inkjet printer can be further classified, based on the type of printhead, into thermal inkjet and piezoelectric inkjet printer. These can also be classified into drop-on-drop or drop-on-solid on the basis of the substrate on which the printhead deposits the drops (22).

#### Thermal Inkjet Printer (TIJ)

**Fig. 1:**
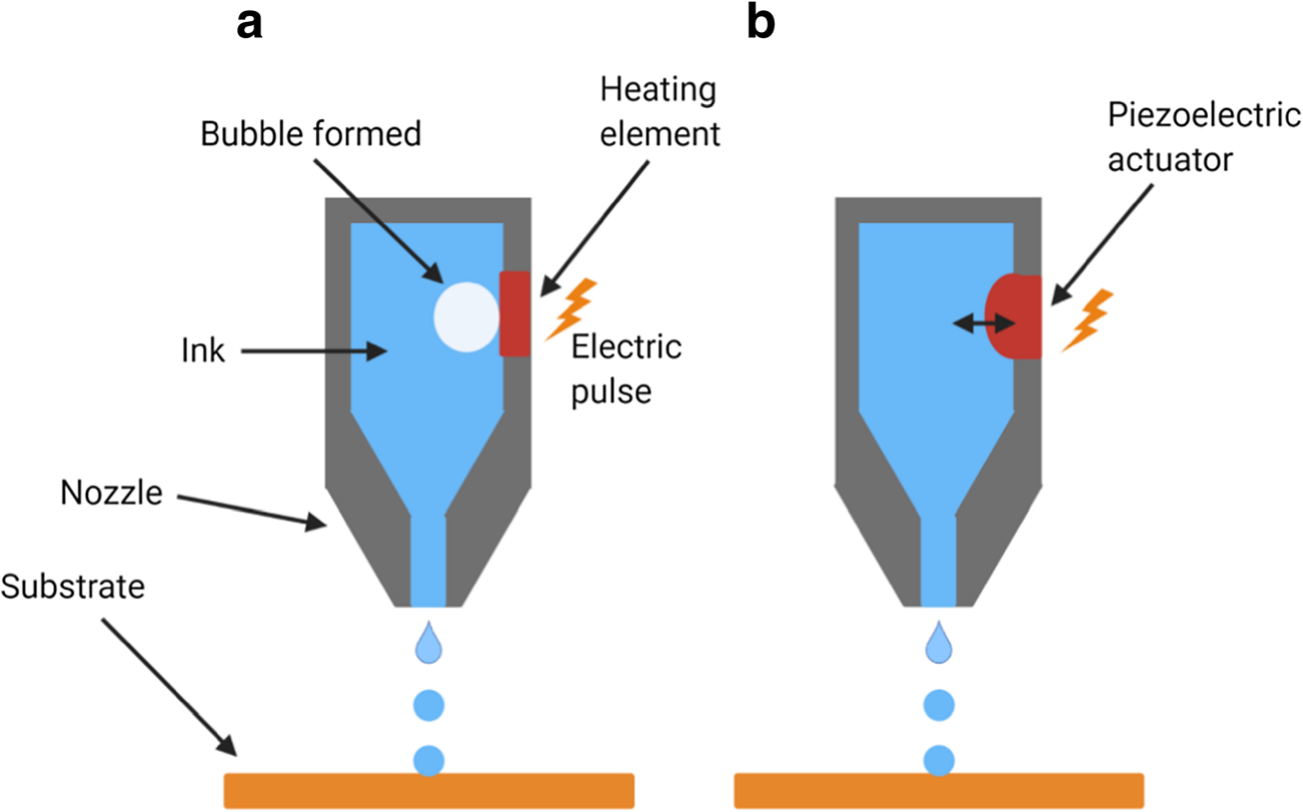
Schematic of inkjet printhead. **a** Thermal. **b** Piezoelectric

Here, thermal energy is the trigger mechanism used to discharge droplets, which then exit the nozzle. The printheads have resistors embedded in them which are in immediate exposure with the fluid (ink) and which upon induction of electric current produce heat. This heat then results in the formation of a bubble within the volatile fluid, which then expands and ejects a small volume of fluid out of the nozzle forming a droplet (Fig. 1a). The main limitation of this technique is the use of high temperatures (200–300°C) of the resistor, which might lead to the degradation of thermo labile active ingredients (18,23).

#### Piezoelectric inkjet printer (PIJ)

This technology composes of a piezoelectric element or actuator which changes its shape in response to an electric voltage. This generates a pressure, which leads to the fluid (ink) being ejected out of the nozzle. After the element gets back to its actual shape, the nozzle is reloaded with the fluid and is ready to be activated again (21,24) (Fig. 1b). The main advantages of this technique include its operability at room temperatures using less volatile and more biocompatible fluids (23).

Another interesting technology called “valve jet” or “electromagnetic” printing was used in pharmaceutical printing, which was based on miniature solenoid valves. This is advantageous due to its robustness and larger orifice sizes compared with thermal inkjet printer (TIJ) or piezoelectric inkjet printer (PIJ) and would help in printing coarser suspensions (25,26).

A glass inkjet tool was developed to eject droplets at high frequencies. It would widely suit pharmaceutical applications as glass being inert will not react with any of the materials used (27).

The inkjet technology can be taken further by combining it with UV photo-initiation. UV curing has been used to harden materials rapidly on demand in the inkjet printing industry. The ink used here contains cross-linking functional groups which get triggered by light and a photo-initiator is often used to promote this process (28).

#### Pharmaceutical Applications of Inkjet Printing

One of the main applications of inkjet printing in pharmaceuticals is in the preparation of orodispersible film (ODF) formulations. They are single sheets or multilayered, made up of appropriate materials having drugs loaded on to them, which liberate the drug rapidly in the mouth to form a solution or suspension in the saliva without chewing or water consumption (29). Thabet *et al*. used PIJ printing to print enalapril maleate onto hydroxy propyl cellulose–based ODFs which were either drug free or contained hydrochlorothiazide. Water- or methanol-based inks were used for this purpose. The doses of enalapril and hydrochlorothiazide could be adjusted in enalapril on hydrochlorothiazide films to obtain various fixed dose combinations (30). TIJ was used to develop ODFs of propranolol hydrochloride in a mixture of water and glycerol on three different edible substrates, *i.e*., rice paper, coated rice paper, and icing sheet. Saccharin was used as a sweetener to improve palatability, which was included using a casting knife (31). A novel approach of dosing two drugs simultaneously and independently on ODFs using TIJ printer was discussed by modifying a commercial TIJ printer. T3 (liothyronine sodium) and T4 (levothyroxine sodium) were printed on hydroxypropyl methyl cellulose substrates and ink solutions were prepared in mixtures of ethanol, DMSO, and propylene glycol (PG) (32).

Mucoadhesive buccal films were also developed using TIJ in combination with fused deposition modeling (FDM). Here, ibuprofen ink was deposited on HPMC films prepared by FDM technology (33). Another oromucosal dosage form was developed using PIJ, where lidocaine hydrochloride was printed on electrospun gelatin substrates with or without piroxicam (34). Hence, the combination of two technologies to develop pharmaceuticals was demonstrated to be effective. Most of these films used for oral delivery face the disadvantage of limited ink and, hence, drug loading efficiency. To overcome this, edible solid foams were developed which were porous and appropriate for inkjet printing of higher amounts of ink (35). Apart from the deposition of small molecules, there has been research in printing biologics on a suitable substrate using inkjet printer for buccal delivery (36,37).

Inkjet technology has also found its usage in transdermal delivery. Films for transdermal delivery were developed by PIJ technology which were used to load ink formulations of indomethacin in ethanol on polythene films (38). Inkjet printing has also been used to coat microneedles for transdermal delivery (39–41).

Dropwise additive manufacturing of pharmaceutical products (DAMPP) which uses drop-on-demand technology has been developed to manufacture various dosage forms (20). Using this technology, self-emulsifying drug delivery systems (SEDDS) have been developed to increase the solubility of drugs. Icten *et al*. developed a formulation using DAMPP by placing a film of a self-emulsifying mixture on a tablet and polymer-based films (42).

Apart from these formulations, tablets of carvedilol and ropinirole have been prepared by using inkjet with photo-initiation (28,43). Also, solvent inkjet printing was used to prepare tablets of thiamine hydrochloride (44). Other formulations developed using inkjet technology were aerogel microspheres for pulmonary delivery (45) and drug-loaded mesoporous silica nanoparticles (46).

### Binder Jet Printing

**Fig. 2:**
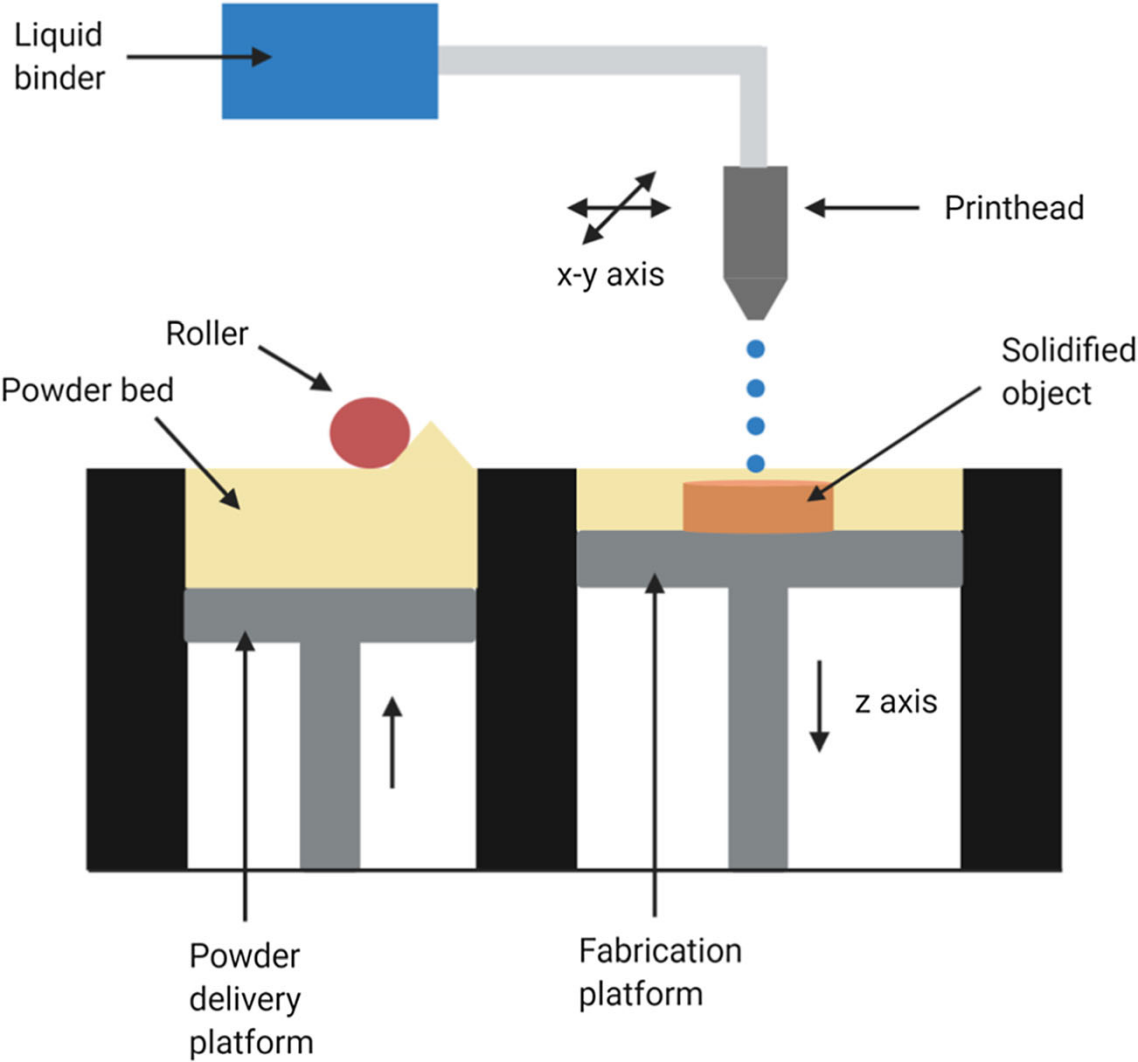
Schematic of binder jet printer

Binder jet printing, also known as drop-on-powder method is an application of inkjet printing technology. The printhead of a binder jet printer can either be thermal or piezoelectric. It composes of a powder bed which is fused in a layer wise fashion. A printer nozzle which contains the binder (and/or drug) fluid is programmed to jet the liquid onto the loose powder bed by moving along an *x*-*y*-axis. The powder is in turn moistened by the liquid drops which lead to hardening and solidification of the layer. The powder solidification occurs either by forming binder bridges or by the dissolution and re-crystallization of particles. The fabrication platform then moves downwards across the *z*-axis, and the powder delivery platform moves up. A roller then moves a powder layer from the bed to the top of the formerly bound layer. This procedure is repeated successively and the 3D object is constructed (Fig. 2). The object is then procured from the powder bed and the unattached powder removed. Thermal sintering is often used to remove any residual volatile solvent. FDA approved Spritam was developed using the zip dose technology, which is based on binder jetting (15). Another technique based on binder jetting is the TheriForm method which is a novel microfabrication process used to construct dosage forms layer by layer, one layer at a time (47).

#### Pharmaceutical Applications of Binder Jet Printing

There has been a lot of research on the applications of binder jetting for the fabrication of tablets. The type and concentration of excipients used in the tablet manufacture process by binder jetting have a great impact on the tablet characteristics. It was demonstrated that filling agents having high water solubility, moistening agent having high water content and binders with high viscosity in solution can enhance the hardness and binding strength of the tablets and extend their disintegration time (48). Another study which demonstrated hydroxy propyl cellulose as a potential binder, concluded that the tablet friability vastly depended on the particle size of the binder used (49). Another study investigated the use of linear and 4-arm star polyvinyl pyrrolidone as the binder. They concluded that the compressive strength of a tablet depended on the weight percentage of polymer in the binder. Since 4-arm star polymers had lower viscosities compared with the linear analogues, they were jettable at higher concentrations, producing stronger tablets. Also, the inclusion of acetaminophen in the tablets showed adequate physical properties at 5–50% concentration in each tablet (50).

Tablets of various APIs with different shapes and release profiles have been developed using binder jetting. Tablets of chlorpheniramine using binder solutions of Eudragit E-100 and Eudragit RLPO were developed, using ethanol and acetone as the solvents, respectively. The tablets were printed with six layers of placebo in the bottom, followed by eight layers comprising of the active ingredient, which was then followed by six more placebo layers on top (51). Another study developed dosage forms of captopril using the TheriForm process, using mannitol as the bulk excipient along with maltitol, maltodextrin or polyvinyl pyrrolidone as powder additives (47). Apart from that, tablets of pseudoephedrine (52), acetaminophen (53), 5-fluorouracil (54), and amitriptyline hydrochloride (55) were developed using binder jetting.

### Fused deposition modeling (FDM)

**Fig. 3:**
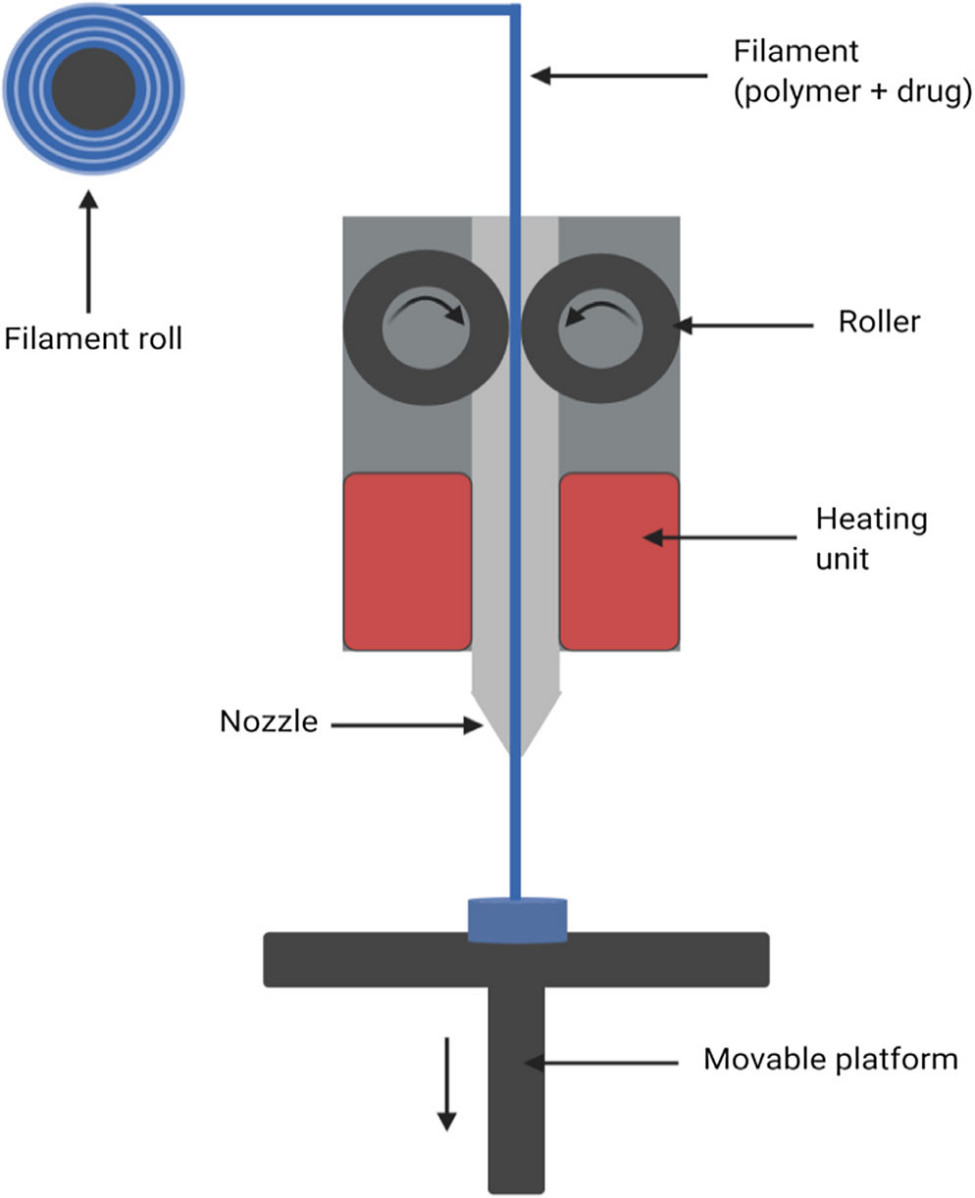
Schematic of fused deposition modeling

Fused deposition modeling (FDM) or fused filament fabrication is the most extensively used 3D printing technique. Thermoplastic drug–loaded polymeric filaments, which after developing, are fed into the printer, where they are melted at a specific temperature and extruded through the nozzle. The printhead moves in a rastor platform and the extruded filament is unloaded on the printer platform, creating the first layer of the object. Successive layers are then deposited upon lowering the platform each time to allow room for the subsequent layer. The filaments cool down and attach to the preceding layer. This procedure is repeated to produce the final 3D article (Fig. 3). The printhead temperature can be manipulated in most printers allowing the use of various polymers and polymer blends (56).

The filaments used for FDM are mostly prepared by the hot melt extrusion (HME) process, where the drug is incorporated into the polymer along with various excipients. This process uses a screw-based extrusion system in a barrel, which is driven by a motor and uses heat and pressure to melt the mixture, which is then left to cool down. This mixture then hardens to produce the filament which is to be used as the feed for FDM (56,57). The wide use of FDM in pharmaceuticals is attributed to its cost-effectiveness, printing accuracy, guaranteed quality parameters and the incorporation of HME (58).

Direct powder 3D printing (DPP), which is a one-step FDM process without HME was researched on. Here, the powder blends after loading into a stainless steel extrusion cartridge, were heated and successfully printed to fabricate tablets with a honeycomb design (59).

#### Pharmaceutical Applications of FDM

There has been a plethora of research on FDM”s capabilities to develop various pharmaceutical products (Table I). Tablets of various shapes and release profiles (60,64,66), bilayer tablets (69,70), and tablet in device systems (77) were fabricated. Sustained release capsule shaped tablets (Caplets) of theophylline were developed (78). Development of capsules (87) and multi-compartmental capsules (88) were also carried out.

**Table I:** Pharmaceutical Applications of FDM

Dosage form	Active pharmaceutical ingredient (API)	Polymers	Special characteristics	Reference
Tablets	Tramadol	Hydroxypropyl cellulose (HPC), polyethylene oxide (PEO)	Modified release, abuse deterrent	(60)
	Bicalutamide	Kollicoat IR	Modified release	(61)
	Dronedarone HCl	Polyethylene glycol (PEG), polyvinyl alcohol (PVA)	-	(62)
	Metformin HCl	PVA	Egg-shaped tablet—Egglet, abuse deterrent	(63)
	Isoniazid	HPC, Hydroxy propyl methyl cellulose, PEO, Eudragit, Kolliphor	Modified release	(64)
	Rebamipide	Hypermellose phthalate	Controlled drug release	(65)
	Metformin HCl	PVA	Ethanol-water (9:1) increased drug loading	(66)
	Carvedilol, haloperidol	PVA	Rapid drug release	(67)
Osmotic tablets	Diltiazem	Core—PVA Shell—cellulose acetate	Shape of CA varied which modified release	(68)
Bilayer tablets	Enalapril maleate, Hydrochlorothiazide	Eudragit	Dynamic dose dispensing	(69)
	Metformin, Glimepiride	Eudragit, PVA	Combination of two release profiles	(70)
Gastro-retentive tablets	Cinnarizine	HPC, vinyl pyrrolidone-vinyl acetate copolymer (Kollidon)	Controlled release	(71)
	Theophylline	HPC	Controlled release	(72)
Gastro-retentive floating devices	Acyclovir	Polylactic acid (PLA)	Tablet in device, controlled release	(73)
	Theophylline	HPC, ethyl cellulose	Tablet in device, pulsatile drug release	(74)
	Baclofen	PLA	Tablet in device, sustained release	(75)
	Amoxicillin	PVA	Capsule in device, prolonged drug release	(76)
	Riboflavin	PLA, poly caprolactone (PCL)	Tablet in device, controlled release	(77)
Caplets	Theophylline	HPC, Eudragit, PEG	Sustained release	(78)
Fast dissolving oral films	Paracetamol, Ibuprofen	PEO, PVA	Single-layered or multilayered films with circular, square, and mesh shape	(79)
Mucoadhesive buccal films	Diclofenac sodium	PVA	-	(80)
	Ibuprofen	HPMC	Drug loading was done by inkjet printing	(33)
Microneedles	Fluorescein	PLA	-	(81)
Suppository shell	Progesterone	PVA	Holes were made in the shell	(82)
Meshes	Ciprofloxacin	Polypropylene, PVA	-	(83)
Orthodontic retainers	Clonidine HCl	PLA, PCL	Local sustained release of drug	(84)
Anti acne mask	Salicylic acid	Flex Eco PLA and PCL	Nose-shaped masks	(85)
Vaginal rings	Progesterone	PLA, PCL	Controlled release	(86)

In the last few years, there has been extensive research in the use of FDM in gastro-retentive dosage forms, from tablets (71,72,89) and capsules (90) to capsular devices (73,75,76) and pulsatile drug delivery systems (74). Apart from these, oral fast dissolving films (79) and mucoadhesive buccal films (80) were also fabricated. FDM was used to fabricate substrates for the deposition of drugs onto them by inkjet printing (33). Other pharmaceutical applications include microneedles (81), drug containing orthodontic retainers (84), drug containing topical masks (85), and drug-loaded vaginal rings (86). The pharmaceutical applications are summarized in Table I.

### Selective Laser Sintering (SLS)

**Fig. 4:**
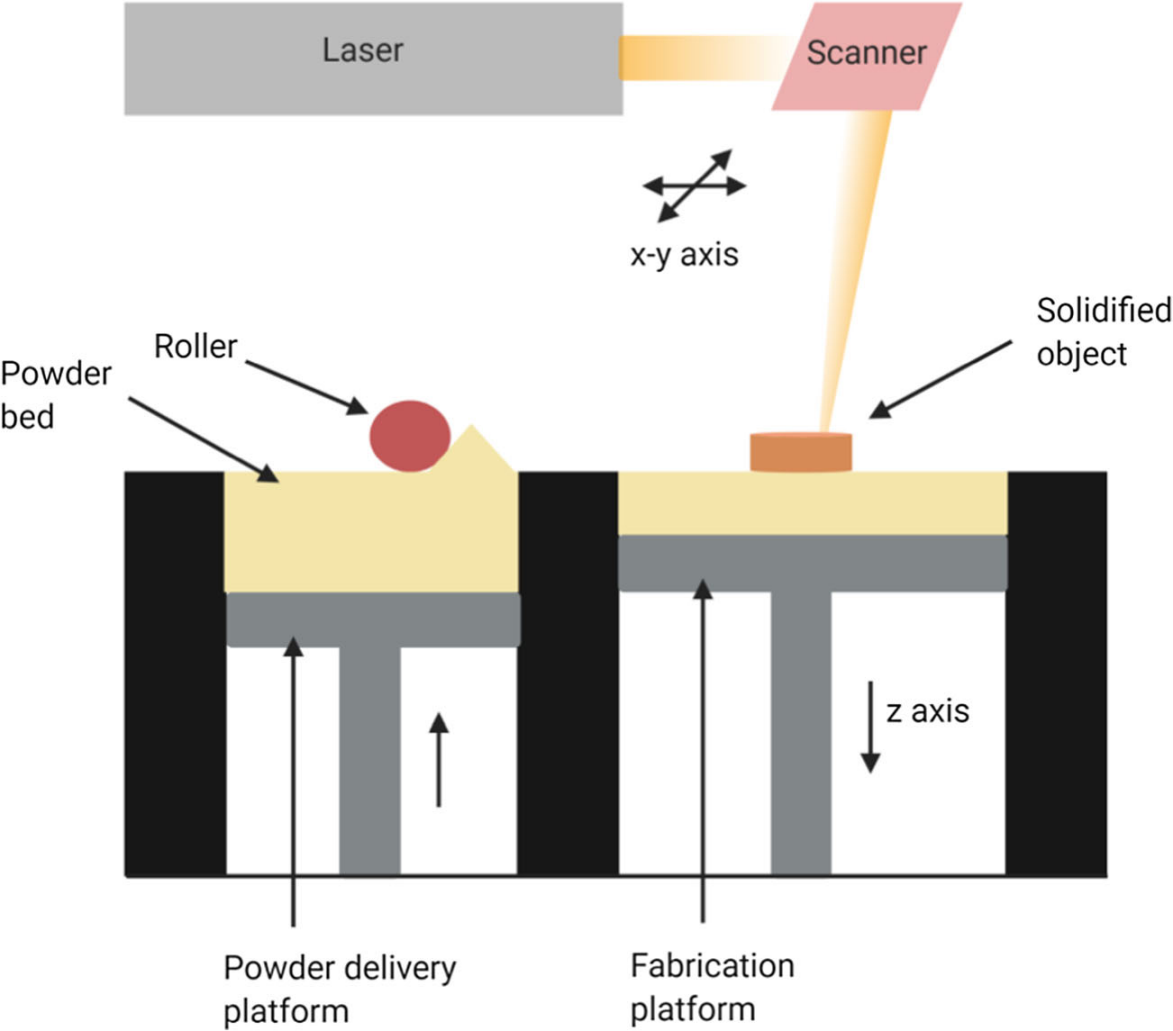
Schematic of selective laser sintering

Selective laser sintering employs laser energy to heat and fuse the particles of powder, which solidify to form a 3D object. The main components of the selective laser sintering (SLS) system are spreading platform, powder bed, and laser system. The spreading system first spreads the powder uniformly on the platform and a roller blade is used to even the surface. The scanning pattern of the laser system, which moves in a 2-dimensional plane, is predetermined based on the properties of the finished product. The material is heated to a temperature below its melting point to cause fusion by melting using laser, and the height of the bed is altered to center laser on the freshly formed surface. The loose powder on the platform provides support during the process. Each time, the powder bed is moved down by a height of one layer and the next layer is deposited and fused. This is repeated to construct the final 3D printed object, which after cooling inside the printer, is gathered from the loose powder manually or by using a sieve (91) (Fig. 4). This method is advantageous as it is a one-step fast production process and does not use any solvent. It also produces objects with high resolution due to the laser precision (92).

#### Pharmaceutical Applications of SLS

SLS is not so widely used in the production of drug-loaded formulations due to the high energy laser, which might degrade the drugs (92). Various delivery devices for drug loading using SLS have been explored (93,94).

Recently, development of oral drug-loaded formulations using SLS has been researched. 3D printed tablets (printlets) of paracetamol were developed using two polymers, namely Kollicoat and Eudragit, which showed no evidence of drug degradation (92). Orally disintegrating printlets of paracetamol using polymers HPMC and kollidon were fabricated (95). Printlets of ondansetron were used for the complexation after incorporation into cyclodextrin with mannitol and kollidon (96). Various mini-printlets combining paracetamol and ibuprofen with customized drug release patterns were also investigated (97). Paracetamol-loaded gyroid structures were also developed using polymers polyethylene oxide, Eudragit and ethyl cellulose (98).

### Stereolithography (SLA)

**Fig. 5:**
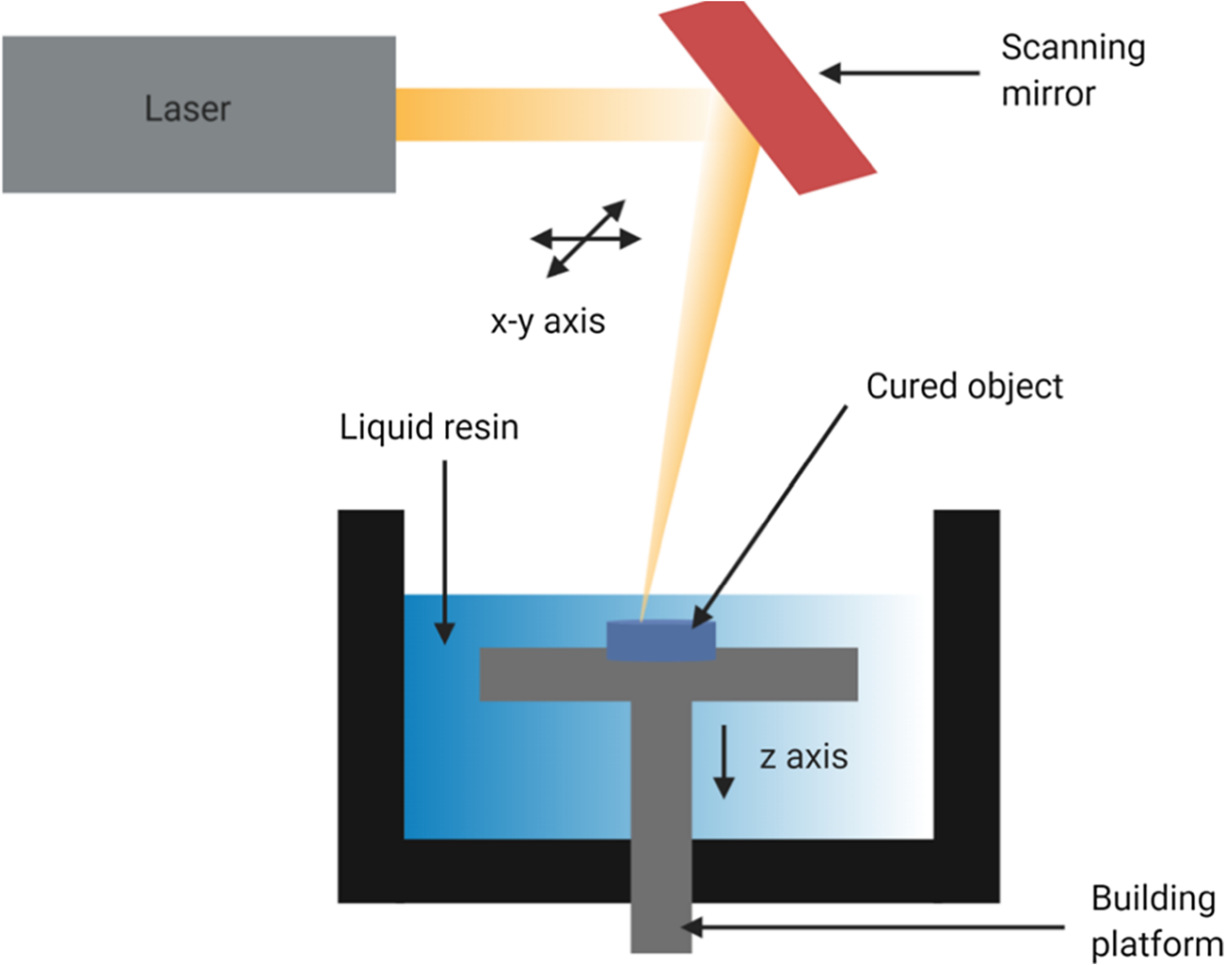
Schematic of stereolithography

Stereolithography is based on the hardening of liquid resin by photo-polymerization using ultraviolet light. The set-up of the printer can either be bottom-up, where the UV source is located below the printer and the moving platform above, or top-down, where the UV source is above and the platform is below. The first layer, after being traced by the laser in the x and *y*-axis directed by scanning mirrors, gets photo-cured and attaches to the building platform. The platform then moves across the *z*-axis to an extent which depends on the width of each layer (moved down in case of bottom-up approach and elevated in case of top-down approach). Following this, the liquid resin is redistributed above the previously hardened layer for its hardening and the process is continued to build the 3D object (Fig. 5). The object is then cleansed with alcohol to get rid of the excess resin. Post-curing can be employed using a UV oven to strengthen the object (22,99).

The materials used for SLA must have photo-curable characteristics in order to undergo photo-cross-linking. The advantages of this printing process include high resolution and low thermal stress involved.

#### Pharmaceutical Applications of Stereolithography

Despite its advantages, this printing process has a limited application in the pharmaceutical sector. One reason being the smaller number of adequate polymers for pharmaceutical use, none of which have been listed as generally recognized as safe (GRAS) substances. Hence, they are not suitable for human use and also have stability issues due to their photosensitivity. Another limitation is that the photo-initiator fragments might get trapped in the photo-polymerized structures, which upon leaching out of the structure can prove to be cytotoxic (99). Also, one of the studies found an unexpected chemical reaction, *i.e*., a Michael addition reaction between the photo-polymer and drug (100).

Wang and team successfully employed SLA printing to construct paracetamol tablets with modified release profiles using polyethylene glycol di-acrylate as the monomer and diphenyl (2,4,6-trimethylbenzoyl) phosphine oxide as the photo-initiator (101). It was also used to develop paracetamol tablets of various shapes to obtain different release profiles (102).

Hydrogels have also been developed using this technology. Martinez and team prepared hydrogels loaded with ibuprofen consisting of cross-linked polyethylene glycol di-acrylate. It was shown that hydrogels that contained and retained water could be printed by the addition of water to the resin formulation and this is because water can be trapped in the matrices (103). Ascorbic acid–loaded solid dosage hydrogels were also fabricated using the polymer poly(ethylene glycol) di-methacrylate and riboflavin as the photo-initiating substance (104).

Various microneedles for transdermal delivery were also fabricated, which were then coated by the drug using inkjet printing (39,105). A novel, hybrid manufacturing process was developed for drug delivery systems incorporated with drug depots, where the matrix of the DDS was generated by SLA and loading of drug depots was done using inkjet printing (106). Microreservoirs for implantable and transdermal delivery were developed (107). Goyanes and associates developed salicylic acid–based anti-acne masks using FDM and SLA. In the SLA prepared masks, mixtures of PEGDA and PEG were used. SLA was concluded as the superior method due to higher resolution, higher loading of drug and the absence of drug degradation (85).

### Pressure assisted microsyringe (PAM)

**Fig. 6:**
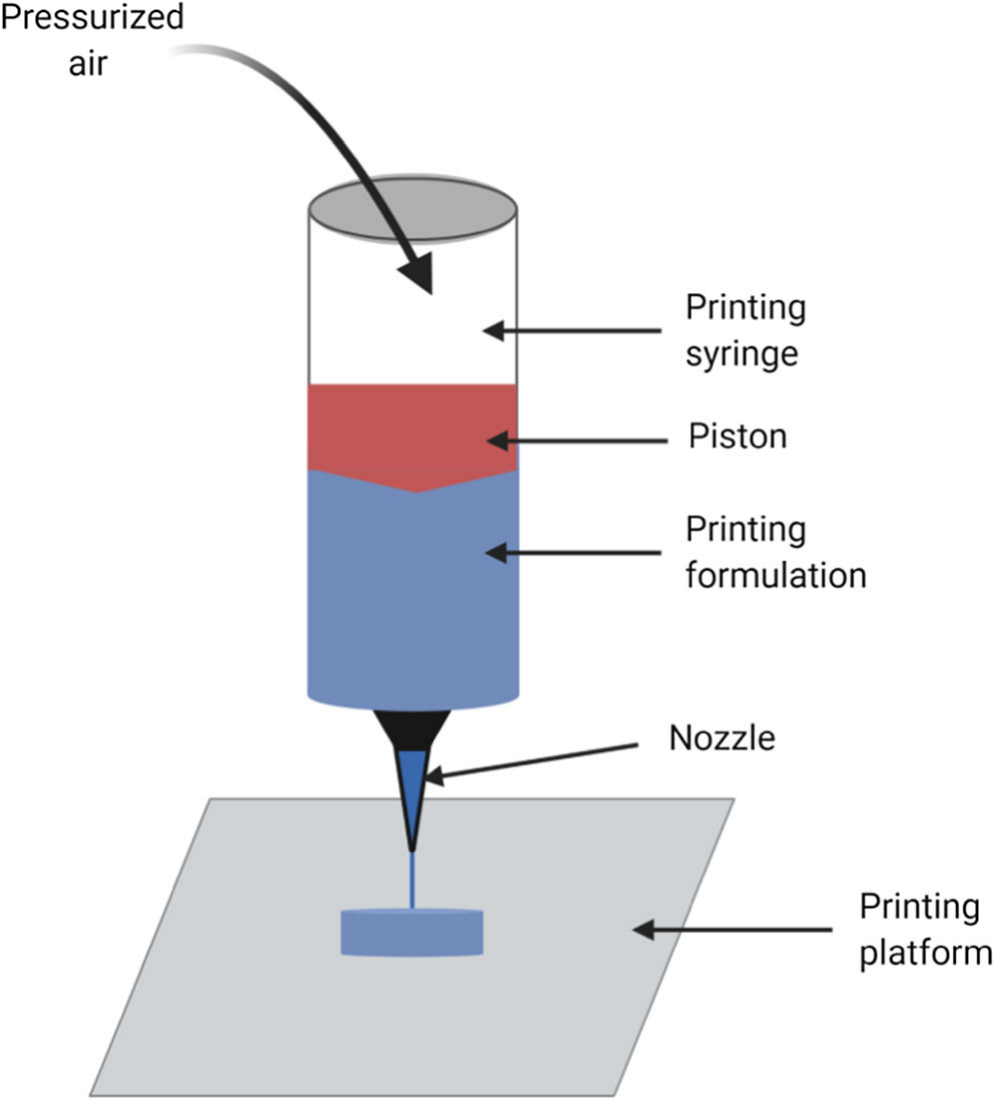
Schematic of pressure-assisted microsyringe

Pressure-assisted microsyringe involves a syringe-based head fed with semi-solid material from which the material is extruded continuously, in a layer upon layer fashion, to construct a 3D printed object (Fig. 6). The extrusion can be based on a mechanical, pneumatic or solenoid piston. The semi-solid mixture consists of a suitable mixture of polymer, solvent, and additional necessary excipients that have properties suitable for printing. Drying after the printing process becomes crucial because of the use of solvents, which might in turn lead to shrinking or deformation of the final product. Also, if the deposited layer does not strengthen enough to overcome the weight of the succeeding layers, the printed object might collapse. The main advantage of this process is the absence of high temperatures (108).

#### Pharmaceutical Applications of Pressure-Assisted Microsyringe

Aita *et al*. used pressure-assisted microsyringe (PAM) to develop immediate release tablets of levetiracetam which were free of organic solvents. Polyvinyl alcohol-polyethylene glycol graft co-polymer was employed as the matrix, and the effect of another polymer, polyvinyl pyrrolidone-vinyl acetate co-polymer on the tablets was determined. The tablets with additional PVP-PVAc showed increase in dissolution and disintegration time (109). The same team then developed tablets using polyvinyl acetate/polyvinyl pyrrolidone co-polymer, HPMC and highly dispersed silicon dioxide where the dissolution profiles could be altered by modifying the amount of HPMC (110).

HPMC-based gastro-retentive tablets of ginkgolide were prepared using PAM. Lactose and microcrystalline cellulose were used to form a homogenous paste, to increase the formability (111). Mucoadhesive oral films of HPMC loaded with catechin were prepared using hydrogel-based printer inks (112).

Table II shows a brief comparison of the various 3D printing technologies, along with their advantages and disadvantages. Table III summarizes some studies under each technology from the literature.

**Table II:** Comparison of Various 3D Printing Technologies

Method	Material	Advantages	Disadvantages
Inkjet	Ink—drug solution Substrate—polymer-based films	• Continuous inkjet—prevents clogging of nozzle • Drop-on-demand inkjet—high precision, low cost and minimizes wastage of material	• Continuous inkjet—wastage of material, low resolution, and expensive • TIJ—might degrade heat sensitive materials
Binder jet	Binder fluid, powder bed	• Can be performed at room temperature • Use of a wide range of starting material • Fast disintegrating dosage forms can be produced	• Post-fabrication drying becomes necessary • Use of organic solvents • Wastage of powder material • Produces fragile dosage forms
Fused deposition modelling	Thermoplastic polymeric filaments	• Comparatively low-cost process • Solvent-free process • No post-fabrication steps • Produces mechanically strong dosage forms	• Heat involved might degrade certain material • Polymers used must be thermoplastic • Prior preparation of filaments necessary
Selective laser sintering	Laser energy absorbing powder	• One step fast production process • Solvent-free process • Produces objects with high resolution	• Only laser energy absorbing components can be used • High energy laser might degrade drugs
Stereolithography	Photo-curable liquid resin	• High-resolution process • Low thermal stress involved	• Starting materials must have photo-curable characteristics • Post-curing steps necessary • Less number of polymers approved for pharmaceutical use • Stability issues while storing photo-sensitive resins • Products can be cytotoxic
Pressure-assisted microsyringe	Semi-solid mixture of polymers and solvents	• Room temperature process • Wide range of starting material can be used	• Use of organic solvents • Post-fabrication drying necessary

**Table III:** Some 3D Printing Studies from the Literature

Method	Year	Dosage form	Drug/biologic	Objective	Outcome	References
Inkjet	2017	Scaffold	Lidocaine hydrochloride, piroxicam	To study the use of electrospun fibrous substrates for the fabrication of inkjet-printed dosage forms	• Good entrapment and solidification of the drug in the matrix • Printed drug amount in good correlation with theoretical dose	(34)
	2018	Buccal film	Lysozyme	To study the effects of printing biologics on films in terms of their physical and mucoadhesive properties	• Printing successful without affecting mechanical or mucoadhesive properties	(36)
	2019	Transdermal patch	Indomethacin	To study the application of PIJ in the development of indomethacin films	• Patches loaded with indomethacin alcoholic solution showed crystalline structure, whereas the addition of PVP showed amorphous form • Patches showed good drug release and permeation	(38)
	2020	Buccal film	Ketoprofen, lidocaine hydrochloride	To fabricate and evaluate buccal films by combining FDM and inkjet printing	• Alterations in the characteristics of films observed • Mucoadhesive and mechanical properties effectively modified • Films showed good release and lack of any toxicity	(113)
	2020	Buccal film	Thiamine hydrochloride, nicotinic acid	To fabricate and evaluate buccal films comprising of B-complex vitamins	• Inkjet printing did not affect the solid state of the matrix • Burst release of both vitamins within 10 min • Higher concentrations of vitamins onto the substrate improved *in vitro* permeation	(114)
Binder jet	2018	Tablet	Caffeine	To evaluate various HPC grades as solid binders in the powder formulation to print tablets	• HPC was a suitable binder for binder jetting • Tablet friability depended on the particle size of the binder • Disintegration and dissolution time depended on the viscosity of the binder	(49)
	2020	Tablet	Amitriptyline hydrochloride	To fabricate tablets using binder jet printing and evaluate the drug loading efficiency of the printing process	• The minimum drug loading was 30 μg • Tablets had a porous surface • They showed immediate release	(55)
	2020	Tablet	Indomethacin	To study various powders and liquid binders as possible materials for binder jet printing	• A moulding method was developed to select appropriate powder and binder materials • The polymer Kollidon produced tablets with a higher breaking force • Disintegration time of the printed tablets were shorter than their moulded counterparts • Breaking force of the printed tablets limited by the amount of binder jetted • Spraying water onto the tablets after printing increased the breaking force	(115)
FDM	2017	Orodispersible film	Aripiprazole	To fabricate and evaluate orodispersible films of aripiprazole	• PVA was concluded to be a suitable polymer for orodispersible film fabrication • Amorphization of aripiprazole was achieved which led to increase in dissolution rate • Mechanical properties of the printed films were comparable with the casted films	(116)
	2018	Tablet in device	Riboflavin	To design and evaluate a gastric floating system where a tablet is accommodated into a device	• Device showed good floating ability and optimal drug release • Floating time reached more than 3 days	(77)
	2019	Tablet	Pregabalin	To fabricate a floating sustained release system	• Processing conditions did not significantly affect the drug stability • Crystallinity of the drug retained even after printing • Drug release was quicker from an open system with low infill percentage compared with closed systems with high infill percentage • Tablet with partially opened top layer exhibited zero-order drug release	(117)
	2020	Tablet	Paracetamol	To fabricate and evaluate tablets with a prolonged release core surrounded by an insoluble shell to obtain zero order release	• Tablets showed zero order release profiles for 16–48 h • Dose contained in the tablet could be infinitely adjusted for personalized medicine	(118)
	2020	Tablet	Rufinamide	To improve the dissolution of rufinamide through solid dispersions using 3D printing	• A mixture of HPMC, Kollidon, Soluplus, Triacetin and gelucire were the suitable excipients to improve the dissolution	(119)
	2020	Tablet	Cinnarizine	To fabricate and evaluate a gastro retentive dosage form of cinnarizine	• The tablets floated instantly, and the floating force was constant up to 12 h • Drug release followed zero order kinetics and was controlled from 6 h to ≥ 12 h • Printing parameters and design had a good correlation with tablet performance	(71)
SLS	2017	Tablet	Paracetamol	To investigate SLS printing for the fabrication of tablets using polymers, Kollicoat and Eudragit	• Printed tablets were robust • No evidence of drug degradation observed • Kollicoat formulations showed pH independent release which depended on drug content • Eudragit formulations showed pH dependent release, independent of drug loading	(92)
	2018	Tablet	Paracetamol	To investigate the feasibility of SLS to fabricate orally disintegrating tablets with accelerated release	• Modifying the laser scanning speed of the SLS printer affected the release characteristics and accelerated release tablets were obtained	(95)
	2019	Tablet (Pellets)	Paracetamol, ibuprofen	To produce small oral dosage forms (mini-printlets) with modified release using SLS	• Tablets of 1 and 2 mm diameter were fabricated • Ethyl cellulose–based printlets exhibited prolonged drug release • Dual printlets with customised release patterns were obtained by varying the polymer	(97)
	2020	Tablet	Ondansetron	To fabricate and evaluate orodispersible printlets of ondansetron	• Tablets fabricated disintegrated at 15 s and > 90% drug released within 5 min	(96)
	2020	Tablet	Clindamycin Palmitate hydrochloride	To fabricate and evaluate clindamycin printlets by SLS	• Laser scanning speed, amount of microcrystalline cellulose and lactose monohydrate affected tablet properties • Decrease in crystallinity of lactose monohydrate observed in printlets	(120)
SLA	2017	Hydrogel	Ibuprofen	To fabricate and evaluate ibuprofen-loaded hydrogels of cross-linked polyethylene glycol diacrylate	• Hydogels containing up to 30% w/w water were prepared • Hydrogels with higher water content released the drug faster	(103)
	2018	Microneedles	Insulin	To fabricate and evaluate polymeric microneedle patches for transdermal delivery of insulin	• Pyramid and cone shaped microneedles were fabricated which were coated using inkjet printing • Insulin integrity and stability was preserved • Insulin was released within 30 min irrespective of microneedle design	(39)
	2019	Microneedles	Cisplatin	To fabricate and evaluate polymeric microneedle arrays for the delivery of cisplatin	• Excellent piercing ability observed with 80% penetration • Good release rates and anti-cancer activity observed	(105)
	2019	Tablet	Paracetamol, Aspirin	To fabricate and evaluate tablets of two drug concentrations (2.5% and 5%) using SLA	• 28 dosage forms were successfully fabricated in one print process • Sustained release over 24 h observed • Statistically significant differences were observed from target range, indicating scope for process improvement	(121)
	2020	Hydrogel	Ascorbic acid	To employ SLA to create custom hydrogel tablet geometries using a water soluble and novel resin	• Poly(ethylene glycol) dimethacrylate–based polymer network was employed to create various geometries • Honeycomb and coaxial annulus tablet gels showed highest release	(104)
PAM	2018	Tablet	Levetiracetam	To fabricate and evaluate tablets using polyvinyl alcohol-polyethylene glycol (PVA-PEG) copolymer and checking the effects of another polymer polyvinyl pyrrolidone-vinyl acetate (PVP-PVAc) copolymer on the properties of the tablet	• Tablets with PVA-PEG showed immediate release within 10 min • Addition of PVP-PVAc showed slight delay • Tablets with PVA-PEG disintegrated faster than PVP-PVAc	(109)
	2018	Tablet	Ginkgolide	To develop and evaluate a gastro-retentive, controlled release formulation	• Tablets had good mechanical properties • Effect of various printing parameters on the tablet was studies	(111)
	2020	Tablet	Levetiracetam	To develop formulations for PAM which are free of organic solvents and have a good long-term stability	• Drug release could be modified by varying the amount of HPMC and by changing the infill design • Tablets had a high structural integrity, uniform content and mass	(110)

## POTENTIAL OF 3D PRINTING IN PERSONALIZED MEDICINE

One of the major potentials of 3D printing in the pharmaceutical sector is its ability to tailor the dosage forms to individuals. This can be done by fabricating adequate dosage forms, adjusting the doses, combining them or by varying the release profiles of the dosage forms according to the need of patients.

### Dose Personalization

3D printing can offer potential in achieving flexibility of doses according to the patient needs. One major population group that calls for dose flexibility is the pediatric group in which the therapeutic dose varies according to the age and body weight of children. Various dosage forms mentioned above can be adequately modified using 3D printers to dispense the best dose for patients. In ODF formulations, this can be easily done by modulating the amount of liquid API dispensed on the film. ODFs can also be subjected to changes in shape and dimension to individualize treatments (29). Likewise, dose strength can be modified in other dosage forms like tablets or patches to tailor them for patient needs. For example, Pietrzak *et al*. used FDM and HME to print theophylline tablets of doses varying from 60 to 300 mg by manipulating the printing scale (122).

In the past years, tablet splitting by hand or by using a splitter, has been undertaken to achieve dose flexibility. This has been proven to be ineffective as the various characterization parameters of the subdivided tablets do not always comply with the Pharmacopoeial standards. Zheng *et al*. carried out the research where split tablets were compared with 3D printed subdivided tablets. The 3D printed subdivided tablets were concluded to be more accurate, safe, and had the potential for customization (122). 3D printed pellets or mini-printlets, which are mini-tablets, have been developed, and these can be personalized. They can also be used to combine two different drugs (97). Mini-printlets can also be combined and encapsulated according to the dose necessary to achieve personalization (123).

### Modifying Release Profiles

3D printing can be employed to obtain dosage forms of various release profiles which can be tailored to individual requirements. One of the techniques to attain this is by altering tablet shapes and geometries. Immediate release tablets of a low dose drugs were fabricated, where it was concluded that by decreasing the tablet thickness or by generating spaces in them, the drug release rates increased and complete release was achieved at times as low as 5 min (124). Khaled *et al*. developed paracetamol tablets of various geometries, namely ring and mesh, and compared them with each other and solid tablets. Immediate release was achieved with the mesh tablets, whereas ring and solid tablets demonstrated sustained release (125).

In another study, paracetamol tablets of the shapes cube, disc, sphere, pyramid, and torus were fabricated. The study concluded that the drug release form the printlets can be modified by altering the surface area/volume ratio (102). Tablets of complex geometries like that of a honeycomb structure were fabricated using 3D printing. The honeycomb cell sizes were varied from 0.20 to 1.83 mm to obtain various release profiles. It was concluded that dosage form geometries could be manipulated to achieve different release profiles (126).

Tablets of complex release profiles which combined two different release mechanisms were also fabricated. This included immediate-extended release tablets with two sections of varying pH-based release mechanisms. Breakaway tablets comprising of three sections, *i.e*., two drugs containing subsections which eroded within 45 min in an environment mimicking the GIT, separated by an interior fast eroding section, were fabricated. Enteric dual pulsatory tablets were also constructed, which showed two pulses of release at 1 and 8 h. Also, dual pulsatory tablets were developed with two sections of opposing pH-based solubility in which one section eroded in the acid dissolution stage within 30 min and the second one began eroding 5 h later at the higher pH stage (51).

Apart from solid tablets, shape was also varied for 3D designed patches. Three shapes of fish gelatin–based polymer hydrogel patches, *i.e*., cylinder, torus and gridlines, were developed which showed variable drug release (127). Also, optimized capsular devices were fabricated, which were used to control drug release from an immediate release tablet inside them, while being suspended in the gastric fluid (75).

Gioumouxouzis *et al*. fabricated osmotic 3D printed dosage forms where the release was altered by altering the shape of the cellulose acetate shell enclosing the osmotic core (68). Another study concluded that in coated tablets, the area of tablet coated, number of coats, and coated sides of the tablets were the parameters which controlled release profile of drugs (128).

Apart from variations in the structure and coating, the excipients used can also moderate drug release by 3D printing. Wang *et al*. used SLA to fabricate tablets of 4-aminosalicylic acid and paracetamol. They concluded that the drug release depends on the percentage of cross-linkable polymers. Higher ratios of poly(ethylene glycol) diacrylate in the tablets decreased the rate of dissolution whereas higher concentration of PEG 300 enhanced the release (101). Tagami *et al*. developed tablets of naftopidil using a semisolid extrusion type 3D printer where hydrogel was used as the printer ink. It was found that increasing the quantity of HPMC in the formulation retarded drug release (129).

### Combination Tablets–Polypills

One of the significant applications of 3D printing in personalized medicine is a concept of “polypill”. A polypill composes of a combination of many drugs in a single tablet which can be tailored for an individual undergoing polypharmacy. Also, the drug release can be tailored as per individual needs. This concept can widely benefit the geriatric populations, where it can improve patient compliance and medication adherence as it reduces the number of pills consumed in a day. Khaled *et al*. successfully fabricated 3D printed polypills with three drugs which could be a possible medication for diabetics with hypertension. These pills compose of an osmotic compartment of captopril and sustained release compartments of nifedipine and glipizide (130). The same team also devised a polypill with five compartments which represented a cardiovascular treatment regimen. The tablet composed of aspirin and hydrochlorothiazide in two immediate release compartments and pravastatin, atenolol, and ramipril in three sustained release chambers (131).

Another team developed PVA-based polypills comprising of four drugs—lisinopril, amlodipine, rosuvastatin, and indapamide—which were explored for multilayer and unimatrix structures. The unimatrix tablets resulted in slower drug release than individual tablets. In the multilayered polypills, the drug release was influenced by its location in the multilayer (132). Martinez *et al*. fabricated multilayered polypills comprising of 6 drugs—paracetamol, prednisolone, aspirin, chloramphenicol, naproxen, and caffeine with different geometries, *i.e*., cylindrical and ring-shaped, using SLA 3D printer. Here, the printer was modified such that the printer could be stopped, resin tray removed, and replaced with different resin solutions (133).

The 3D printing technology was then taken further to develop polypill capsules with varying release profiles for multiple drugs. This was achieved by combining FDM with hot-filling syringes. Two capsule skeletons were designed which had four separate compartments—one with a concentrical configuration with two outer compartments for early release and two inner compartments for retarded release, other with a parallel configuration where non-dissolving capsule shells with free pass corridors and dissolution rate limiting pores were used to achieve early and retarded release. The capsule shell composed of polyvinyl alcohol and polylactic acid. Customized release profiles were obtained through the alteration of shell thickness in the concentric configuration or size of rate limiting orifices in the parallel configuration (134).

### 3D Printing Tending to All Population

#### Tending to Pediatrics

Children seem to be the most difficult of the population to satisfy because of their individual preferences when it comes to the dosage form, taste, shape or smell. Oral delivery might seem to be the most convenient, but might become rather complex when it comes to children. A child may simply reject a dosage form or another because of minor attributes such as preferences in the shape, color or taste. This is where 3D printing can come in and tend to their individual choices (135). A major concern in treating children is the administration of adequate doses according to the body weight, which can be solved using 3D printing, like mentioned above.

Swallowing being a concern in smaller children, ODF formulations, fast disintegrating tablets and mini-pills fabricated by 3D printing seem to be suitable for administration. A study highlighting the dosage form preferences in children concluded that children preferred mini-tablets of 4 mm diameter over other formulations (136). Another study concluded that, in pediatrics, ODF formulations are more suitable than oral powders in unit dose sachets (137).

Medication adherence and compliance in children can be increased by giving them dosage forms in a flavor and color of their choice. Goyanes *et al*. conducted a study where they used 3D printing to fabricate chewable tablets of isoleucine for the treatment of maple syrup urine disease (MSUD). Apart from printing the tablets in different doses as per the requirements, they successfully printed tablets in various flavors (lemon, coconut, banana, raspberry, *etc.*) and colors (yellow, black, light green, orange, *etc.*) which were well accepted by pediatric patients according to their preferences (138). Another study successfully fabricated children-friendly chewable chocolate–based dosage forms in various shapes (139).

#### Tending to Geriatrics

In most of the geriatric population, swallowing tablets becomes a major challenge which can influence medication adherence as swallowing difficulties increase with advancing age (140). This can be resolved by using fast disintegration tablets and oro-dispersible film formulations which can be fabricated by 3D printing as mentioned above.

The older population suffers from multiple ailments and requires multiple drugs and prolonged medication which brings in the issues of polypharmacy (140). Polypharmacy can be resolved by the means of poly-pills fabricated according to the patient’s need by 3D printing. Some of them also suffer from cognitive impairment (dementia) which can affect medication adherence. This can be resolved by using 3D printed dosage forms with embossing designs on them which can indicate the time of administration, date, and/or weekday for administration, customizable to each patient (141).

#### Tending to the Visually Impaired

Visual impairment affects about 285 million people worldwide and this leads to several concerns when it comes to medication and treatment, especially in the older population who are dependent on multiple medications. This leads to poor medication adherence and treatment management which would eventually lead to therapeutic inefficiency. Awad *et al*. used SLA to fabricate orally disintegrating tablets well suited for visually challenged patients. These printlets composed of Braille and Moon patterns on their surface which can enable patients to identify the medication when extracted from their package. Tablets of various shapes were developed which offered added information like medication indication or dosing regimen. This innovative concept can aid the treatment of visually impaired patients greatly by improving medication adherence and reducing medication errors (142).

#### 3D Printing in a Clinical Setting

Studies have shown that 3D printed dosage forms have been well accepted by patients. Most individuals held positive views on medicines printed by 3D printers (138,141,143). In the near future, this technology can have a massive role to play in hospitals and hospital pharmacies to dispense personalized pharmaceutical products. This involves complete diagnosis and fabrication of dosage forms that will be most suitable to patients. In a possible setup, a patient upon his/her arrival at the hospital would undergo diagnosis to identify and analyze his/her medical condition. Other factors will be taken into account like the person’s age, body weight, presence of other conditions, treatment history, and lifestyle. The genetic aspects would also be taken into consideration through body fluid tests. All of this data could be combined to form the individual’s profile which would contain all necessary details. Based on this profile, a treatment plan could be identified, and the patient would receive a prescription which is solely based on his/her needs. The most adequate dosage forms could then be identified using various databases and artificial intelligence platforms. The dosage form could then be printed using a 3D printer present in the hospital, through CAD software. This medication, after suitable quality control, can then be given to the patient. The patient is then followed up and feedback obtained. With technological advances in testing, data processing, and 3D printing, this entire process can be made faster which can prove to be promising in a clinical setting.

## CHALLENGES

Despite its immense benefit in the pharmaceutical sector, 3D printing possesses several challenges. They are mostly regarding the technology, manufacture of dosage forms, safety, quality control, regulatory aspects, and their implementation in clinical pharmacy.

### Technology

The technological limitation of each type of 3D printer has been mentioned above. The nozzle-based systems might suffer from clogging of the nozzle whereas thermal and laser-based systems might suffer from degradation of the API employed. A major challenge is the drug-excipient incompatibility which has to be addressed. Also, structural and surface imperfections may arise in the final product which has to be addressed by optimizing various manufacturing parameters.

### Materials Used

Another challenge may be the availability of suitable materials of appropriate grades for 3D printing, for example, suitable polymers for FDM like HPC, PVA, and Eudragit and resins for SLA like polyethylene glycol di-acrylate and poly(ethylene glycol) di-methacrylate*.* These materials, apart from being compatible with the drug, must be biocompatible, biodegradable and suitable for manufacturing using 3D printing (22). Also, they must not generate toxic substances while processing.

### Safety Aspects

Safety considerations must also be addressed. There is a possibility of emission of toxic airborne matter resulting from the heating, extrusion or fusion of certain materials which can act as respiratory or skin irritants. Hence, adequate safety measures must be taken and standard operating guidelines must be followed to minimize the hazardous exposure (144).

### Clinical Pharmacy Practice

Integrating 3D printing into hospitals also presents several challenges. First of all, it requires highly skilled technical operators on site to handle the technical aspects, which might seem impractical. Another challenge is quality control of the printed dosage forms and techniques for this must be developed which must be non-destructive and feasible. This has already been addressed by involving various process analytical technologies (PAT) to monitor the quality. A PAT model involving near-infrared spectroscopy and Raman confocal microscopy was demonstrated which showed high accuracy in measuring the drug concentration and drug distribution in tablets and oral films (145).

Also, financial aspects must be considered, as establishing 3D printers in hospitals might be expensive. Packaging and labeling requirements must also be considered to meet the requirements of personalized medicine in a clinical setting. Furthermore, since every printing platform mentioned above has their pros and cons, it cannot be concluded as to which type of printer can be ideal for a hospital setting. Hence, further technological developments are necessary to bring in “the perfect 3D printer” for clinical use, which must be fast, user friendly, and cost-effective with a good resolution.

### Regulatory Aspects

Another major obstacle in using this technology for the manufacture of pharmaceutical products is the lack of regulatory framework. In 2017, FDA issued the guidance comprising of regulatory requirements for the manufacturing of medical devices (146). So far, there are several FDA approved 3D printed medical devices in the market, but only one FDA-approved 3D printed pharmaceutical product (Spritam) is available. Unfortunately, guidelines for 3D printed dosage form manufacturing have not been issued by any regulatory authority. Furthermore, it remains unclear if the regulatory approval will apply to only the final product or to a set of requirements which will apply to all components and stages of designing and manufacturing of a product (144).

In the present scenario, a tablet containing more than one drug would be considered as a new combination drug formulation, according to the FDA, and would require extensive clinical trials to guarantee patient safety and efficacy. Also, every location employing a 3D printer for producing and dispensing pharmaceutical products would have to be certified as a “Good Manufacturing Practice” (GMP) facility (23). Hence, appropriate regulatory guidelines must be developed regarding the manufacture and dispensing of pharmaceutical products.

### Anti-counterfeiting

Another major challenge with the advent of 3D printing is that, due to the lack of regulations, there can be a rise in counterfeit medications. These fraudulent medicines often do not meet the minimum quality requirements. Also, they could be obtained easily, at low costs. These medications are not safe for consumption and may bring about harm and additional complications to the people. According to the World Health Organization, 10.5% of low and middle income countries are inflicted with low-grade and falsified medicines which cost an approximate of US$30.5 billion annually. Hence, adequate measures to prevent these fraudulent activities must be taken.

One study developed a novel track and trace anti-counterfeit measure for 3D printed medicines, using inkjet printing. Quick response (QR) codes and data matrices were printed on the surface of the 3D printed printlets, which could be scanned using a smartphone. They were designed to encode tailored information related to the drug product, patient and prescriber. Further, by increasing the number of excipients and coloring agents within the inks used, the randomized code could cover millions of combinations assisting improved tracking and authentication system of pharmaceutical products (147).

## 3D BIO-PRINTING

Yet another wide application of 3D printing is 3D bio-printing. 3D bio-printing involves dispensing cell-loaded biomaterials for the fabrication of complex functional living tissues or organs. It has been used in regenerative medicine to construct tissues like skin, bone, and cartilage. Hence, a different technical approach compatible with the deposition of living cells becomes necessary. Some advantages include scalability, accurate control of cell deposition, high resolution, personalization, and cost-effectiveness (148). Bio-inks are formulations that compose of cells and may include biomaterials and biologically active components. The cells may be in various forms and environments and might even be seeded into various carriers.

Cartilage structures were fabricated using nano-fibrillated cellulose having shear thinning properties and alginate with a good cross-linking ability. These proved to be non-cytotoxic and had good cell viability (149). Bioactive bone scaffolds to enhance bone regeneration were developed, which were based on poly-lactic acid. These were coated with gels comprising of mucic acid. This coating improved their physicochemical properties and favored osteoblast differentiation (150). Another team fabricated small diameter blood vessels with two distinct cell layers using 3D bio-printing. Good cell proliferation and angiogenesis were observed in the blood vessels (151). 3D bio-printing has also been used in the fabrication of neuronal tissues (152). Researchers have also successfully printed a heart using human cells, complete with blood vessels, ventricles, and chambers (153). Despite its benefits, bio-printing does face several problems like cell viability and the control of cell proliferation. The materials and cells employed must also be compatible with the printing process. Another major concern of bio-printing is regarding its safety. The materials used should be biocompatible and safe (154).

## CONCLUSION

The rapid advancement of 3D printing technologies could move the pharmaceutical production from mass manufacture to on-demand personalized dosage forms which would provide patients with more safe and effective medicines. Its potential to transform the conventional pharmacy practice could be pivotal to the healthcare system. Apart from its clinical use, this technology can also be employed in the industries to develop dosage forms with complex shapes and release profiles. The approval of Spritam by the FDA is a remarkable milestone in the field of 3D printing and there has been an abundance of promising research ever since. The greatest advantages of using 3D printing in the pharmaceutical sector include fast manufacturing speed, cost-effectiveness, and formulation flexibility. Furthermore, the pros and cons of various platforms must be analyzed to develop a 3D printer ideal for a hospital setting.

Despite its benefits and considerable progress, the use of this technology to fabricate pharmaceutical products still remains in its infancy. This is due to the various challenges faced, mainly the technical, quality control, and regulatory aspects. Moreover, relevant regulatory guidelines have to be formulated pertaining to the use of this technology in a clinical setting. Once these challenges are addressed, there will be no looking back, and the pharmaceutical sector can completely embrace the technology. It can then be possible to look at a smart future with personalized medicine which can revolutionize the healthcare system.
